# Mechanism for the acute effects of organophosphate pesticides on the adult 5-HT system

**DOI:** 10.1016/j.cbi.2015.12.014

**Published:** 2016-02-05

**Authors:** Sarah J. Judge, Claire Y. Savy, Matthew Campbell, Rebecca Dodds, Larissa Kruger Gomes, Grace Laws, Anna Watson, Peter G. Blain, Christopher M. Morris, Sarah E. Gartside

**Affiliations:** a Medical Toxicology Centre, Newcastle University, Newcastle upon Tyne, NE2 4AA, UK; b Institute of Neuroscience, Newcastle University, Newcastle upon Tyne, NE2 4HH, UK

**Keywords:** Organophosphate, Pesticide, Serotonin, 5-HT, Anxiety, Depression, AChE, acetylcholinesterase, AMPA, α-amino-3-hydroxy-5-methyl-4-isoxazolepropionic acid, DMSO, dimethyl sulfoxide, DNQX, 6,7-dinitroquinoxaline-2,3-dione, DRN, dorsal raphe nucleus, 5-HT, 5-hydroxytryptamine, OP, organophosphate

## Abstract

The neurotransmitter serotonin (5-HT) is involved in mood disorder aetiology and it has been reported that (organophosphate) OP exposure affects 5-HT turnover. The aim of this study was to elucidate the mechanism underlying OP effects on the adult 5-HT system. First, acute *in vivo* administration of the OP diazinon (0, 1.3, 13 or 39 mg/kg i.p.) to male Hooded Lister rats inhibited the activity of the cholinergic enzyme acetylcholinesterase in blood and in the hippocampus, dorsal raphe nucleus (DRN), striatum and prefrontal cortex. Diazinon-induced cholinesterase inhibition was greatest in the DRN, the brain's major source of 5-HT neurones. Second, acute *in vivo* diazinon exposure (0 or 39 mg/kg i.p.) increased the basal firing rate of DRN neurones measured *ex vivo* in brain slices. The excitatory responses of DRN neurones to α_1_-adrenoceptor or AMPA/kainate receptor activation were not affected by *in vivo* diazinon exposure but the inhibitory response to 5-HT was attenuated, indicating 5-HT_1A_ autoreceptor down-regulation. Finally, direct application of the diazinon metabolite diazinon oxon to naive rat brain slices increased the firing rate of DRN 5-HT neurones, as did chlorpyrifos-oxon, indicating the effect was not unique to diazinon. The oxon-induced augmentation of firing was blocked by the nicotinic acetylcholine receptor antagonist mecamylamine and the AMPA/kainate glutamate receptor antagonist DNQX. Together these data indicate that 1) acute OP exposure inhibits DRN cholinesterase, leading to acetylcholine accumulation, 2) the acetylcholine activates nicotinic receptors on 5-HT neurones and also on glutamatergic neurones, thus releasing glutamate and activating 5-HT neuronal AMPA/kainate receptors 3) the increase in 5-HT neuronal activity, and resulting 5-HT release, may lead to 5-HT_1A_ autoreceptor down-regulation. This mechanism may be involved in the reported increase in risk of developing anxiety and depression following occupational OP exposure.

## Highlights

• Organophosphate exposure inhibits dorsal raphe nucleus cholinesterase activity.
• Organophosphate oxon exposure activates 5-HT neurones in the dorsal raphe nucleus.
• Nicotinic and AMPA receptors mediate the oxon-induced activation of 5-HT neurones.
• Organophosphate exposure attenuates the response to 5-HT1A autoreceptor activation.

## 1 Introduction

Organophosphate (OP) chemicals are commonly used as domestic and agricultural pesticides. For example, diazinon (Dimpylate) is used in sheep dip and in dog and cat flea collars, and chlorpyrifos is a very commonly used insecticide for crops. OPs are neurotoxic and some such as sarin are used as chemical weapons. High level OP exposure irreversibly inhibits acetylcholinesterase (AChE), the enzyme responsible for the breakdown of acetylcholine. The resulting acetylcholine accumulation causes hypercholinergic symptoms. Clinical symptoms are generally observed when brain AChE is inactivated to 20–50% of normal activity, less than 10% activity results in seizures, paralysis, and respiratory failure [20]. Regulations are in place to prevent poisoning but there is some evidence that lower OP levels that do not result in substantial toxicity can still have long term health consequences. Thus, some studies have reported occupational OP exposure increases the risk of developing symptoms related to anxiety and mood disorders [21], [26], [31], [37]. However, other studies have reported no association between occupational OP exposure and neuropsychology [9], [30], [34]. These inconsistencies may be due in part to the limitations of retrospective human exposure studies, in particular the lack of comprehensive exposure data and adequate control subjects [29]. Therefore, the potential association between OPs and psychiatric symptoms remains a contentious issue and, without a plausible mechanism of action, it is unlikely to be resolved.

The neurotransmitter 5-HT (serotonin) and associated proteins are heavily implicated in both the aetiology and treatment of mood and anxiety disorders. The majority of 5-HT neurones in the brain are located in the dorsal raphe nucleus (DRN) [39] and these neurones project to frontal brain regions including the prefrontal cortex, caudate putamen and hippocampus [14]. 5-HT release in the DRN and in the projection areas is largely dependent on the firing activity of the DRN neurones, which is regulated by excitatory receptors, including α_1_-adrenoceptors and AMPA/kainate receptors, and inhibitory receptors, including the 5-HT_1A_ autoreceptor [1], [35], [15], [13].

Previous studies examining the effects of acute OP exposure on the adult 5-HT system have focussed mainly on 5-HT levels and turnover in brain homogenates. They reported that acute OP exposure affects 5-HT turnover [27], [8], [10], indicating that the 5-HT system has been activated but the mechanism remains unresolved. The aim of this study was to make a detailed functional investigation of the effects of acute low level OP pesticide exposure on the adult rat 5-HT system. Firstly, the effect of acute *in vivo* exposure to the OP diazinon, at doses below the threshold to induce clinical symptoms (<50% cholinesterase inactivation), on cholinesterase activity was determined; brain regions associated with the 5-HT system were assessed. Secondly, the effect of acute low level *in vivo* diazinon exposure on neuronal activity in the DRN was determined *ex vivo* in brain slices. Finally, a neuropharmacological study was conducted in *in vitro* brain slices to further investigate the effects of the OP pesticides, diazinon and chlorpyrifos on 5-HT neuronal activity.

## 2 Materials and methods

### 2.1 Animals

All experiments were carried out in accordance with the UK Animals (Scientific procedures) Act of 1986 and the European Community Council Directive of 24 November 1986 (86/609/EEC). All efforts were made to minimise animal suffering, to reduce the number of animals used, and to utilise alternatives to *in vivo* techniques, if available. Male Hooded Lister rats (Charles River, Kent, UK) were housed in groups in a temperature controlled room (21–24 °C) with 12:12 h light/dark cycle (lights on at 07:00) with *ad libitum* access to food and water.

### 2.2 *In vivo* treatments

After a minimum 5 day acclimatisation period, rats received a single intraperitoneal (i.p.) injection to allow accurate and efficient delivery of diazinon. In the cholinesterase activity study, rats received 0, 1.3, 13 or 39 mg/kg diazinon and in the electrophysiology study rats received 0 or 39 mg/kg diazinon. Doses were selected to cause <50% cholinesterase inactivation (below the threshold to induce overt hypercholinergic toxicity). Diazinon (Sigma–Aldrich, UK) was mixed with ethanol and Cremophor EL to make a suspension and diluted to the appropriate volume with 0.9% saline (final concentration 1% ethanol, 10% Cremophor EL) [4]. The diazinon mixture or vehicle mixture (1% ethanol, 10% Cremophor EL, 89% saline) was administered shortly after preparation (1 ml/kg).

### 2.3 Cholinesterase activity

At 4, 8 or 24 h after treatment animals were overdosed with isoflurane and decapitated. As we did not expect time of collection to significantly affect cholinesterase activity in rats administered with vehicle, all vehicle treated rats were killed at one time point (4 h after injection). Trunk blood was collected into heparinised tubes, diluted 1:25 in cold 0.1% saponin in phosphate buffered saline (PBS) and frozen at −20 °C. Brains were rapidly removed, cut into 3 mm coronal slices, rapidly frozen, and stored at −80 °C until dissection. The hippocampus, DRN, cerebellum, striatum and prefontal cortex were dissected, homogenised in ice cold Tris-buffered saline (pH 7.4), diluted 1:25 in 0.1% saponin, incubated on ice for 10 min and frozen at −20 °C.

Protein concentration in brain homogenates was quantified using a Bradford assay. Briefly, samples (and bovine serum albumin standards) were diluted 1:50 with Bradford reagent and incubated at room temperature for 10 min before absorbance was read at 595 nm. Cholinesterase activity in blood and brain homogenates was quantified using a modified version of Ellmans colorimetric assay [7], [22], [4]. Limits of quantification in blood were 519 and 564 nmol min^−1^ ml^−1^for AChE and butrylcholinesterase, respectively, and in brain homogenate were 23 and 6 nmol min^−1^ mg^−1^ protein for AChE and butrylcholinesterase, respectively (SDs of repeated measurements * 5). AChE and butrylcholinesterase assay precision was <6% (mean coefficient of variance). Blood (diluted a further 1:5 with 0.1% saponin) or brain homogenate samples (10 μl) were added to the wells of a 96 well plate with PBS (110 μl, 0.1 M, pH 7.4). 5,5’-dithio-bis-2nitro-benzoate (99 μl, 0.25 mM, Sigma–Aldrich Company Ltd) as the chromagen and either acetyl(beta-methyl)thiocholine iodide (11 μl, 155 mM; Greyhound Chromatography, UK) or butyrylthiocholine iodide (11 μl, 218 Mm; Sigma–Aldrich Company Ltd) as the substrate were then added. Absorbance of 5’-dithio-bis-2nitro-benzoate was read at 412 nm for 30 min at 5 min intervals (blood 35 °C; brain homogenate 25 °C).

### 2.4 *In vitro* electrophysiology

For the *in vivo* OP exposure study, rats were killed the day after injection (18 ± 1 h) by isoflourane overdose and then decapitated. For the *in vitro* OP exposure study, treatment naïve rats (*n* = 43) were killed by decapitation without prior anaesthesia. Following decapitation, the brain was quickly removed and submerged in oxygenated (95% O_2_/5% CO_2_) sucrose slush (sucrose 200, HEPES 10, MgSO_4_ 7, NaH_2_PO_4_ 1.2, KCl 2.5, NaHCO_3_ 25, CaCl_2_ 0.5, d-glucose 10 mM, pH 7.4). Slice preparation and electrophysiology have previously been described in detail [17]. Briefly, coronal slices of the midbrain (350 μm thick) were cut and placed in an interface perfusion chamber in an atmosphere of humidified 95% O_2_:5% CO_2_. Slices were perfused with oxygenated aCSF (NaCl 124, MgSO_4_ 2.4, KH_2_PO_4_ 1.25, KCl 3.25, NaHCO_3_ 26, CaCl_2_ 2, d-glucose 10 mM, pH 7.4) containing the α_1_-adrenoceptor agonist phenylephrine (3 μM) to evoke spontaneous firing. The aCSF, the 95% O_2_:5% CO_2_ and the bed of the chamber were warmed to 36–37 °C.

Extracellular recordings were made from neurones in the DRN (Bregma – 7.6 to – 8.3). For experiments using *ex vivo* brain slices from animals administered diazinon (*n* = 5) or vehicle (*n* = 5), all neurones encountered were recorded, as OP exposure may have altered the firing rate and regularity. Two to five neurones were recorded from slices from each animal. Following a period of recording of basal firing activity of each neurone, test compounds were applied via the perfusion medium. 5-HT hydrochloride (25 or 50 μM), phenylephrine (10 μM) and AMPA (3 μM) were applied for 2 min. In slices taken from treatment-naïve rats, putative 5-HT neurones (*n* = 84) were identified on their location in the DRN and their electrophysiological characteristics; broad bi or triphasic action potentials firing slowly and regularly. All neurones were tested with 25–50 μM 5-HT. OPs were applied for 10 min (50 μM). As diazinon and chlorpyrifos are activated to diazinon oxon or chlorpyrifos oxon (CPFO) by P450 enzymes *in vivo*, active oxons were also tested. Oxons were applied for 5 min (50 μM) or continuously (100 μM). Mecamylamine hydrochloride (100 μM) and 6,7-dinitroquinoxaline-2,3-dione (DNQX, 10 μM) were applied continuously in the absence and then presence of CPFO (100 μM). The effects of a single OP or active oxon were tested on one putative 5-HT neurone per slice.

Stock solutions (50 mM) of diazinon and chlorpyrifos and their active oxons (Greyhound Chromatography Ltd, UK/Sigma–Aldrich, UK) were made in dimethyl sulfoxide (DMSO) before diluting in aCSF (final DMSO concentration 0.01%). Stock solutions (10 mM) of other drugs were made in aCSF or distilled water except DNQX (DMSO).

### 2.5 Data analysis

Cholinesterase activity in each sample was averaged from replicated wells and corrected for protein content (homogenate). Differences in cholinesterase activity between regions in the vehicle treated group were analysed using one-way analysis of variance (ANOVA). The effects of OP exposure (dose/time since exposure) within and between regions were analysed by expressing activity as a percentage of mean control activity (in blood or brain regions) and using repeated-measures General Linear Models (brain regions and blood as within subject factors (6), dose (4) and time (3) as between subject factors). Significant effects and interactions were investigated further using two-way and one-way ANOVAs followed by *post hoc* Bonferroni (equal variances) or Games–Howell tests (unequal variances). Significance at p < 0.05 is reported.

Action potential firing rate (Hz) and/or regularity (variance in interspike interval) was calculated from a 2 min period at the start of the firing rate record. Action potential characteristics were taken from a waveform average of three action potentials recorded within the first 5 min of encounter. Effects of *in vivo* OP administration were made using either unpaired t-tests, Mann Whitney U tests or repeated-measures General Linear Models (time since application within subject factors, drugs as between subject factors). Effects of *in vitro* compound applications (e.g. OP applied to slice) were analysed by comparing the neuronal firing rate and/or regularity in the 2 min before applications to activity in a 2 min period during or after applications with paired t-tests and expressed as percentage of control.

## 3 Results

### 3.1 Cholinesterase inhibition following *in vivo* diazinon exposure is greatest in the dorsal raphe nucleus

**Fig. 1:**
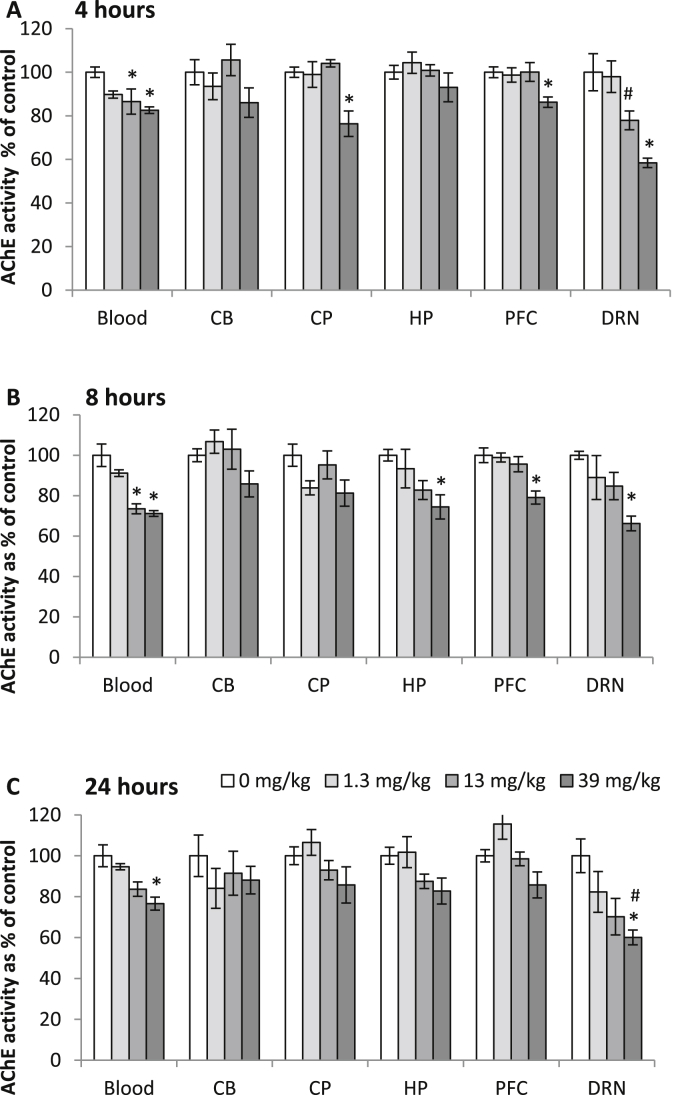
Acetylcholinesterase (AChE) activity in blood and rat brain regions 4 (A, *n* = 8 for each dose), 8 (B, *n* = 6–7 for each dose) and 24 h (C, *n* = 8 for each dose) after acute *in vivo* exposure to diazinon (i.p). AChE activity was affected by dose (F_(3,439)_ = 55.8, *P* < 0.001) and brain region (F_(5,439)_ = 12.8, *P* < 0.001) but not time since exposure with a significant dose*tissue interaction (F_(15,439)_ = 2.9, *P* < 0.001). Exposure to 13 and 39 mg/kg were significantly different to 0 mg/kg (*P* < 0.001) and the dorsal raphe nucleus (DRN) was significantly different to all other brain regions (*P* < 0.001) (Bonferroni *post hoc* tests). *P* < 0.05 compared to all (#) other brain regions (one-way ANOVA with Bonferonni *post hoc* tests for each dose) *P < 0.05 compared to 0 mg/kg (one-way ANOVA with Bonferonni *post hoc* tests for each region). Mean ± SEM. Cerebellum (CB), caudate putamen (CP), hippocampus (HP), prefrontal cortex (PFC).

In samples from vehicle treated rats cholinesterase activity varied between different brain regions (cerebellum, caudate putamen, hippocampus, prefrontal cortex and DRN) (Supplementary Fig. 1A). Acute *in vivo* diazinon exposure caused a small but statistically significant inhibition of blood and brain AChE activity and this was dependent on dose and tissue (blood and brain regions) but not time since exposure (Fig. 1). The dose-dependent effect of diazinon on AChE activity varied between brain regions with no effect in the cerebellum and the greatest effect in the DRN (Fig. 1, Table 1). Acute *in vivo* diazinon exposure also caused a small but statistically significant inhibition of blood and brain butrylcholinesterase activity and this was dependent on dose and tissue (Supplementary Fig. 1B–D, Supplementary Table 1). The dose-dependent effect of diazinon on butrylcholinesterase activity varied between brain regions with no effect in the cerebellum and the greatest effect in the DRN.

### 3.2 *In vivo* diazinion exposure increases neuronal activity in the dorsal raphe nucleus

**Fig. 2:**
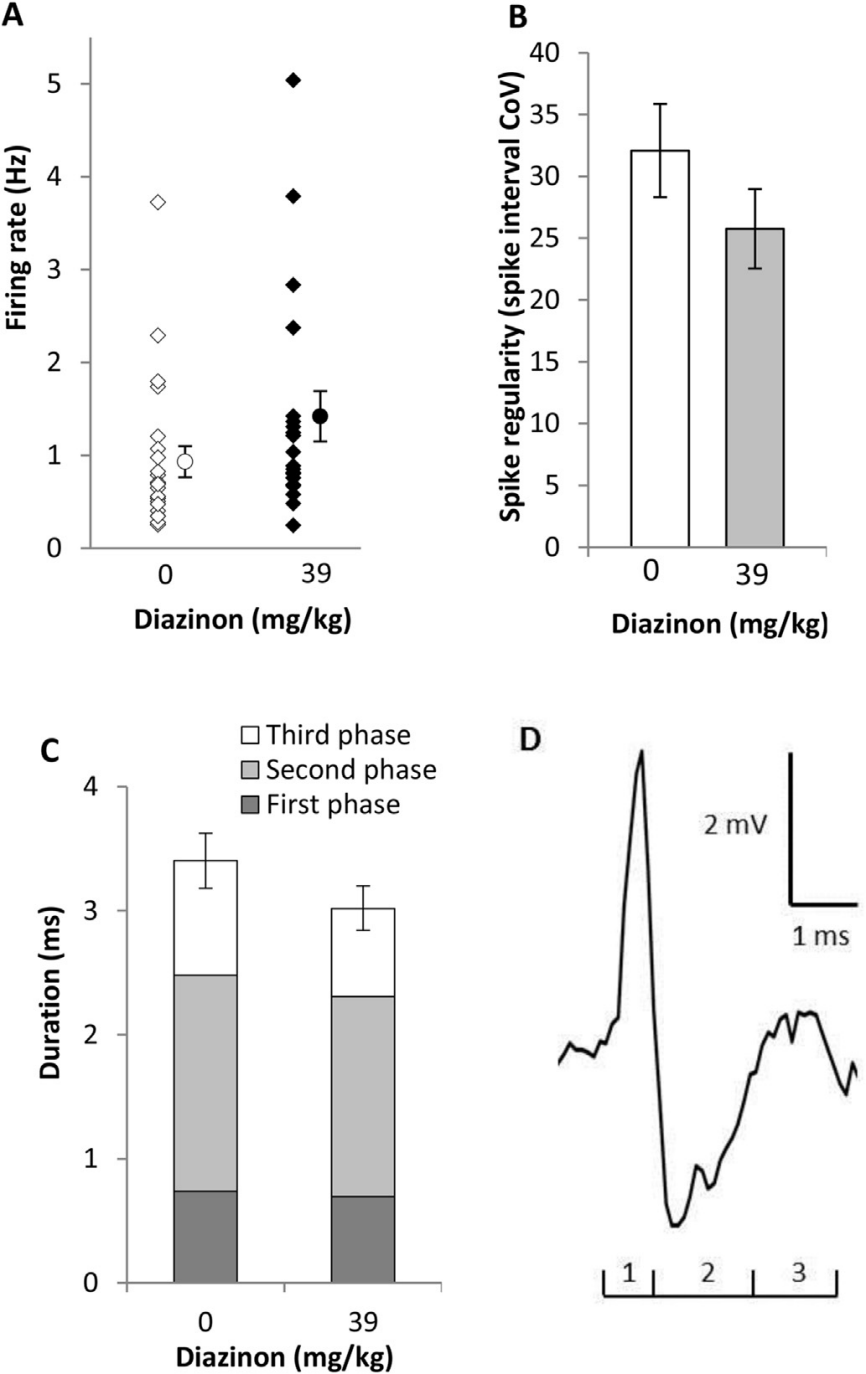
DRN neuronal activity in rat *in vitro* slices one day after acute *in vivo* diazinon exposure. (A) Diazinon exposure increased basal firing rates of DRN neurones (diamonds, individual neurones; circles, mean ± SEM; vehicle *n* = 23, diazinon *n* = 20; U = 144.5, *P* < 0.05). Diazinon exposure did not significantly affect the firing regularity (B) or the mean duration of the three action potential phases (C) of DRN neurones (mean ± SEM). (D) Example of an extracellular recording of a typical action potential from a DRN neurone. The action potential is triphasic (1–3).

To determine if DRN neuronal activity was affected by acute *in vivo* OP exposure, rats were exposed to diazinon (39 mg/kg i.p.) or vehicle and the next day DRN neuronal activity and the sensitivity of receptors modulating firing were investigated *ex vivo* in brain slices. The vast majority of neurones encountered in DRN possessed the general electrophysiological characteristics of 5-HT neurones (firing rate 0.26–3.73 *vs* 0.25–5.04 Hz; interspike interval coefficient of variation 9.8–70.5 *vs* 11.0–63.7; duration 1.7–6.7 *vs* 2.0–5.0 ms; vehicle (*n* = 20) *vs* 39 mg/kg diazinon (*n* = 23). Despite the wide variation in 5-HT neuronal firing rates, neurones from animals exposed to diazinon fired on average 50% faster than DRN neurones from animals exposed to vehicle (0.9 Hz *vs* 1.42 Hz; vehicle *vs* diazinon, Fig. 2A) but their firing regularity and action potential duration did not differ (Fig. 2B–C).

**Fig. 3:**
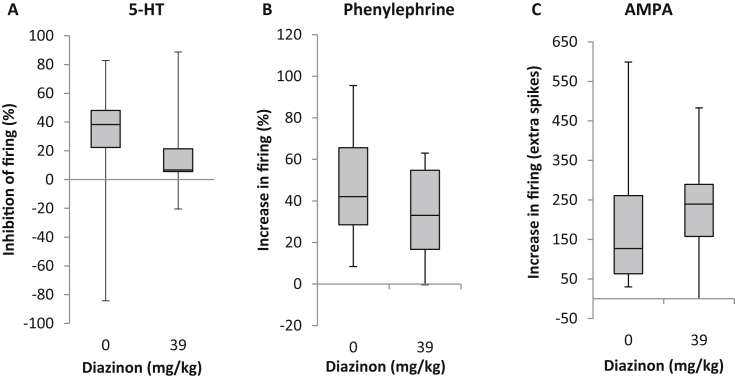
Response of rat DRN neurones in *in vitro* slices to receptor agonists, one day after acute *in vivo* diazinon exposure. (A) *In vivo* diazinon exposure (39 mg/kg i.p.) decreased the inhibitory response of DRN neurones to 2 min applications of 50 μM 5-HT (vehicle *n* = 13; diazinon *n* = 14; U = 48, *P* < 0.05). (B) Excitatory responses to the α_1_-adrenoceptor agonist phenylephrine (10 μM, vehicle *n* = 16, diazinon *n* = 13, *NS*) and to (C) AMPA (3 μM, vehicle *n* = 17, diazinon *n* = 12, *NS*) were not affected by diazinon exposure (quartiles ± min/max).

The vast majority of neurones encountered were inhibited by 5-HT (25–50 μM) although a minority were excited (2/23 vs 3/20, vehicle vs 39 mg/kg diazinon). The response of DRN neurones to 5-HT in the diazinon exposed group was 80% smaller in comparison to the response of the vehicle exposed group (38% *vs* 8% inhibitory response; vehicle *vs* diazinon, 50 μM, Fig. 3A; 25 μM, Supplementary Fig. 2). Excitatory responses of DRN neurones to the α1-adrenoceptor agonist phenylephrine (10 μM) and to AMPA (3 μM) were not significantly different between vehicle and diazinon exposed groups (Fig. 3B–C).

### 3.3 Cholinergic and glutamatergic receptors are involved in the OP-induced activation of 5-HT neurones

**Fig. 4:**
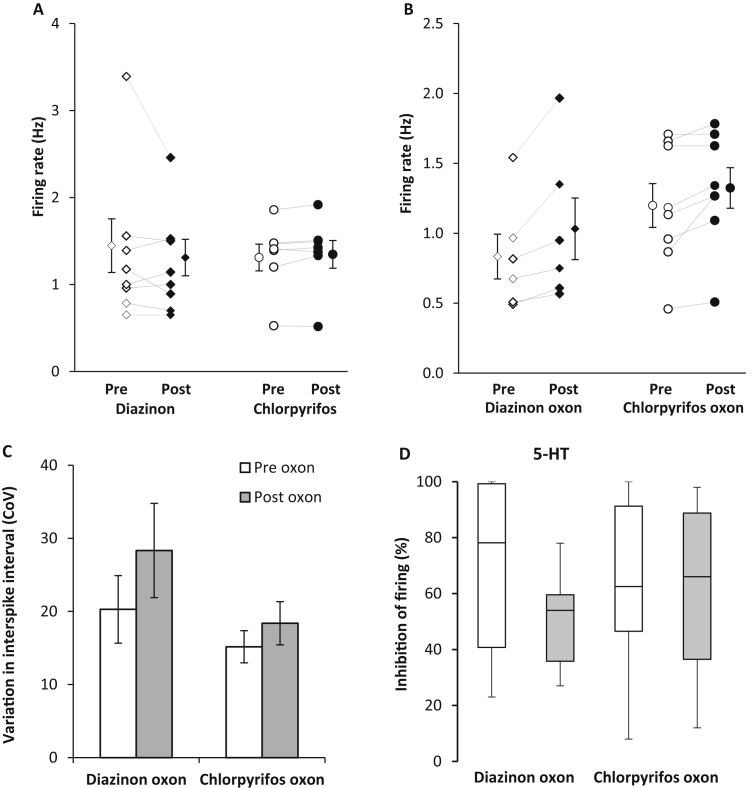
5-HT neuronal activity in rat *in vitro* slices before and after brief application of organophosphate pesticides or their oxon metabolites. (A) Basal firing rate of 5-HT neurones were not affected by the application of diazinon (*n* = 8) or chlorpyrifos (*n* = 6) (*NS*). The firing rates of individual neurones before (white diamond/circle) and during application (black diamond/circle) are shown in addition to the mean ± SEM (B) Application of diazinon oxon (*n* = 6, *P* < 0.05) or chlorpyrifos oxon (*n* = 8, *P* < 0.05) caused a small but significant increment in basal firing rates. (C) Oxon exposure did not significantly affect the firing regularity of the 5-HT neurones (mean ± SEM, *NS*). (D) Inhibitory responses to 5-HT (25 μM) of neurones found before oxon application and the responses of neurones found up to 3 h after application were not significantly different (*n* = 8 diazinon oxon, *NS; n* = 6 chlorpyrifos oxon, *NS;* quartiles ± min/max).

To further understand the effects of OP exposure on DRN neuronal activity, neuropharmacological experiments were conducted in *in vitro* brain slices containing the DRN. The basal firing rate of putative 5-HT neurones in the DRN ranged from 0.48 to 3.64 Hz (1.4 ± 0.7 Hz, *n* = 84) and their inhibitory response to 5-HT ranged from 8 to 100% (55 ± 4%, 25 μM). Brief application of the OP pesticides diazinon (50 μM, 10 min, *n* = 8) or chlorpyrifos (50 μM, 10 min, *n* = 7) to *in vitro* slices did not alter the basal neuronal firing rate of the neurones (Fig. 4A). In contrast, application of diazinon oxon (50 μM, 5 min, *n* = 6) caused a 24% increase (0.83 Hz *vs* 1.03 Hz, before *vs* after, Fig. 4B) and chlorpyrifos oxon a 10% increase in neuronal firing rate (1.2 Hz *vs* 1.32 Hz, before vs after, 50 μM, 5 min, *n* = 8). Firing rate regularity was not significantly affected by oxon application (Fig. 4C) but burst firing (brief periods of firing > 2 × basal firing rate) was observed in 3/6 neurones exposed to diazinon oxon (0/8 neurones tested with chlorpyrifos oxon). The inhibitory responses of 5-HT neurones to 5-HT (25 μM) found before oxon application were not significantly to the responses of neurones found in the same slice up to 3 h after oxon application (Fig. 4C).

**Fig. 5:**
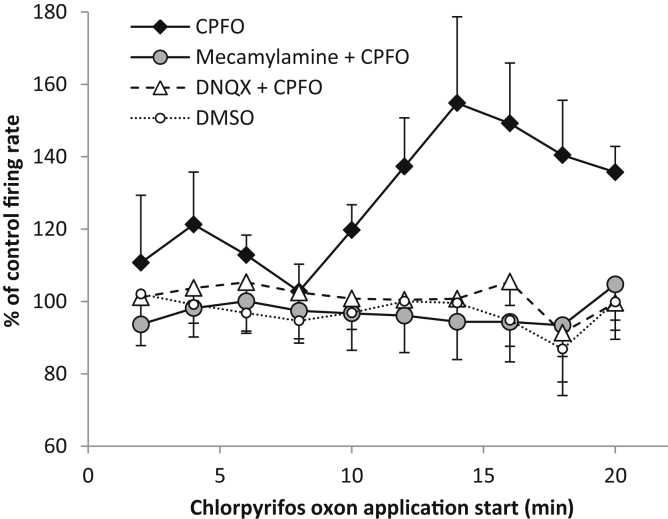
5-HT neuronal activity in rat *in vitro* slices in the presence of chlorpyrifos oxon with and without receptor antagonists. Continuous application of chlorpyrifos oxon (100 μM CPFO) increased the firing rate of 5-HT neurones (*n* = 15). Application of mecamylamine (100 μM, plus 20 min pretreatment, *n* = 7) or 6,7-dinitroquinoxaline-2,3-dione (DNQX, 10 μM, plus 5 min pretreatment, n = 6) with chlorpyrifos oxon blocked the increase in firing rate. Repeated measures ANOVA revealed a main effect of treatment (F_(3,30)_ = 4.0, *p* < 0.05). Oxon application alone was significantly different to oxon application in the presence of mecamylamine (*p* < 0.01) and oxon in the presence of DNQX (p < 0.05, post hoc tests). The vehicle dimethyl sulfoxide (DMSO, 0.01%) did not affected firing rate (*n* = 6). Mean ± SEM.

To investigate the receptors involved in mediating the oxon-induced increase in 5-HT neuronal firing activity, CPFO was applied continuously in either the absence or presence of the nicotinic acetylcholine receptor antagonist mecamylamine or the AMPA/kainate glutamate receptor antagonist DNQX. Continuous application of the vehicle DMSO (0.01%) had no effect on the firing rate (n = 6, Fig. 5). CPFO (100 μM) alone caused a triphasic response in the neuronal firing rate (small increase, decrease and then larger increase, n = 15, Fig. 5). In the presence of either mecamylamine (100 μM, n = 7) or DNQX (10 μM, n = 6) CPFO had no effect on the neuronal firing rate (Fig. 5).

## 4 Discussion

Despite clinical evidence to suggest that the 5-HT system may be affected by OP exposure, this is the first study to demonstrate the functional effects of OP pesticide exposure on the DRN, the major source of forebrain 5-HT in the brain and demonstrate a mechanism of action. Acute exposure to relatively low levels of diazinon caused a small but statistically significant dose-dependent inhibition of AChE and butrylcholinesterase in blood and brain regions associated with the rat 5-HT system. The dose-dependent inhibition of AChE and butrylcholinesterase varied between brain regions with the greatest effect being in the DRN. Acute diazinon exposure also caused a subtle increase in DRN neuronal firing activity and attenuated neuronal inhibitory responses to 5-HT, measured *ex vivo*. Direct application of the oxon metabolites of diazinon and chlorpyrifos to rat brain slices increased the firing activity of DRN 5-HT neurones. The oxon-induced augmentation of firing was blocked by a nicotinic acetylcholine receptor antagonist and an AMPA/kainate glutamate receptor antagonist.

### 4.1 Acetylcholinesterase in the dorsal raphe nucleus is inhibited by organophosphate exposure

Due to differences in route of exposure, the onset of OP-induced AChE inhibition observed in this study is likely to be more rapid than the onset following occupational exposure. Organophosphate absorption through the skin, the primary route of occupational exposure [2], is relatively slow in comparison to intraperitoneal injection or oral exposure [38], [12]. Nevertheless, the effects of diazinon on ChE observed in this study demonstrate that not only is the DRN affected, but the extent and duration of inhibition was greater than in other brain regions examined. The sensitivity of the DRN ChE to diazinon does not appear to be dependent on the basal levels of ChE activity, consistent with previous reports on OP-induced ChE inhibition [32], [36]. These data indicate that OP exposure below the threshold to affect other parts of the nervous system and induce clinical cholinergic symptoms (50% brain AChE activity) could potentially still affect the DRN and 5-HT system.

### 4.2 Neuronal firing in the dorsal raphe nucleus is augmented by organophosphate exposure

The firing activity of individual neurones in the DRN recorded in this study varied widely, consistent with previous studies [16], [15]. Despite this wide variation we report that acute OP exposure (the day before) significantly increased the basal firing rates of neurones in the DRN, as did direct *in vitro* application of the OP oxons. Whilst, the most common neurones in the DRN are 5-HT neurones, other neuronal types are also present. 5-HT-containing neurones are normally identified on the basis of their electrophysiological characteristics [15], [17], [13]. In the *in vivo* OP exposure study, we decided to record all neurones encountered in the DRN from both OP exposed and vehicle exposed animals, as we did not know how OP exposure might affect these characteristics. Thus, it is possible that a minority of non-5-HT neurones were also recorded in this experiment. In the *in vitro* electrophysiological study, 5-HT neurones were identified prior to the application of the OPs or OP oxons, and so we are more confident these were all 5-HT neurones.

Our results indicate that the OP-induced augmentation of DRN neuronal firing activity, and 5-HT system activation, is a consequence of oxon-mediated ChE inhibition, leading to local acetylcholine accumulation. First, ChE activity in the DRN was significantly inhibited by acute OP exposure. Second, direct application of diazinon and chlorpyrifos to 5-HT neurones had little effect on firing activity whereas direct application of their respective oxons did. Most OPs, including diazinon and chlorpyrifos, are poor ChE inhibitors and need to be metabolised to their respective oxons in order to phosphorylate and inhibit ChE activity. Finally, the oxon-induced augmentation of firing could be blocked with a nicotinic acetylcholine receptor antagonist, indicating that it was the consequence of a local acetylcholine accumulation. It has been postulated that the excitatory response of 5-HT neurones to nicotinic receptor agonists, is mediated by nicotinic receptors on the 5-HT neurones [11] and also on local glutamatergic neurones, which release glutamate and thus cause additional 5-HT neuronal excitation via AMPA/kainate receptors [11], [3]. Consistent with this suggestion, we observed that the oxon-induced augmentation of firing could be blocked with an AMPA/kainate receptor antagonist.

### 4.3 5-HT_1A_ autoreceptor regulation of neuronal firing is attenuated by organophosphate exposure

The firing activity of DRN neurones, and 5-HT release in the projection areas, is regulated in part by excitatory receptors, including α_1_-adrenoceptors and AMPA/kainate receptors, and inhibitory receptors, including the 5-HT_1A_ autoreceptors located on the cell bodies in the DRN [1], [35], [15], [13]. We determined that acute OP exposure (assessed one day later) did not alter the response of DRN neurones to α_1_-adrenoceptor or AMPA receptor activation but attenuated the response to 5-HT. The inhibitory response of DRN 5-HT neurones to 5-HT is blocked by the 5-HT_1A_ receptor antagonist WAY100635 [15] indicating it is mediated by 5-HT_1A_ autoreceptors. This OP-induced attenuation of the functional response to 5-HT_1A_ receptor activation is consistent with a previous report that exposure to the OP methamidophos for four weeks, decreased 5-HT_1A_ receptor expression in adult mice [19]. In our study, the 5-HT response does not appear to be affected within the first few hours of direct oxon application, suggesting that oxons do not directly affect the receptors. As with other physiological systems, the 5-HT system is highly dynamic and responds and adapts to change by altering the expression and sensitivity of its components. For example, we have previously found that very low level diazinon exposure (1 mg/kg i.p. for 5 days) decreases 5-HT transporter expression [33]. Thus, we would suggest that the 5-HT_1A_ autoreceptor downregulation reported in this study is a functional adaptation to the increase in 5-HT release following the oxon-mediated increase in neuronal firing.

Adaptations in the 5-HT system following an initial OP insult have previously been reported; 5-HT levels in homogenate change between 4 days and 8 weeks after acute OP exposure [6], [28], [23], [25]. Some have suggested that the OP induced effects on the 5-HT system are independent of ChE inhibition as no overt cholinergic toxicity was observed. Given our results and the sensitivity of the DRN we would argue that, in some cases at least, the changes in components of the 5-HT system may be secondary adaptations to ChE inhibition following low level OP exposure.

It is probable that even subtle changes to the regulation of the 5-HT system as a consequence of low level OP exposure will alter behaviour. Indeed, we have previously reported that low level exposure to diazinon (1 mg/kg i.p. for 5 days) can alter behaviour in rats [33]. Although we cannot be certain that the OP-induced behavioural change was entirely due to alterations in 5-HT system, the anxiety-like behaviour we observed is sensitive to antidepressants which target the 5-HT system [18].

## 5 Conclusion

In humans, changes in anxiety and mood are associated with alterations in the 5-HT system. For example, 5-HT_1A_ receptor binding in the raphe nuclei is reduced by 29% in people with panic disorder [24] and 42% in people with depression [5]. We observed an 80% reduction in raphe nuclei 5-HT_1A_ receptor sensitivity following low level OP exposure. This observation alone does not demonstrate a direct link between OP exposure and psychiatric symptoms but together with the sensitivity of the DRN to OP exposure, the proposed mechanism of action and the results from previous laboratory and epidemiological studies the evidence is consistent with OP exposure increasing the risk of developing anxiety and mood disorders.

## Figures and Tables

**Table 1:** Univariate general linear model test results showing the effect of time and dose of acute diazinon exposure (i.p.) on adult rat acetylcholinesterase activity in different tissues. If there was an effect of dose Bonferonni *post hoc* comparisons were made. *P* < 0.05 in bold.

	Time		Time*Dose		Dose		Bonferonni comparisons with 0 mg/kg, *P*		
*F (df)*	*P*	*F (df)*	*P*	*F (df)*	*P*
						1 mg/kg	13 mg/kg	39 mg/kg
Blood	3.1 (2,74)	**.050**	1.4 (6,74)	0.245	29.3 (3,74)	**.000**	**.021**	**.000**	**.000**
Cerebellum	1.1 (2,74)	0.327	0.7 (6,74)	0.661	2.2 (3,74)	0.094			
Caudate Putamen	1.5 (2,74)	0.231	1.6 (6,74)	0.147	8.5 (3,74)	**.000**	1.000	1.000	**.000**
Hippocampus	6.5 (2,74)	**.002**	1.1 (6,74)	0.399	8.7 (3,74)	**.000**	1.000	0.099	**.000**
Prefrontal cortex	2.9 (2,75)	0.059	1.5 (6,75)	0.175	16.5 (3,75)	**.000**	1.000	1.000	**.000**
Dorsal raphe nucleus	1.5 (2,69)	0.241	0.7 (6,69)	0.667	21.3 (3,69)	**.000**	0.412	**.000**	**.000**
