# Supplementary material for: Mechanism for the acute effects of organophosphate pesticides on the adult 5-HT system
Source: Chem Biol Interact. 2016 Feb 5;245:82–9. doi: 10.1016/j.cbi.2015.12.014 (PMC4732990; doi:10.1016/j.cbi.2015.12.014)
Supplement: Supplementary file 1 [file mmc1.docx]

**Supplementary data**

## Mechanism for the acute effects of organophosphate pesticides on the adult 5-HT system

Sarah J. Judge^a,b1^, Claire Y. Savy^a,b^, Matthew Campbell^b^, Rebecca Dodds^a,b^, Larissa Kruger Gomes^b^, Grace Laws^a,b^, Anna Watson^a,b^, Peter G. Blain^a,b^, Christopher M. Morris^a,b^ and Sarah E. Gartside^b^

^a^Medical Toxicology Centre, Newcastle University, Newcastle upon Tyne, NE2 4AA, UK

^b^Institute of Neuroscience, Newcastle University, Newcastle upon Tyne, NE2 4HH, UK

E-mail: [s.j.judge@ncl.ac.uk](mailto:s.j.judge@ncl.ac.uk)

**Supplementary Figure 1.** Cholinesterase activity in rat blood and brain regions following acute diazinon exposure. (A) Control acetylcholinesterase (AChE) and butrylcholinesterase (BChE) activity (4 hours after exposure to vehicle i.p.) varied significantly between brain regions (repeated measures ANOVA, AChE F_(2,17)_ = 138.9, *P* < 0.001, BChE F_(2,15)_ = 22.0, *P* < 0.001, *n* = 8). Diazinon-induced inhibition of BChE activity in samples collected 4 (B, *n* = 8 for each dose), 8 (C, *n* = 6­–7 for each dose) and 24 hours (D, *n* = 8 for each dose) after exposure (i.p) was dependent on dose (F_(3,63)_ = 17.6, *P* < 0.001) and tissue (F_(4,240)_ = 21.1, *P* < 0.001) but not on time with a significant dose*tissue interaction (F_(11,240)_ = 4.1, *P* < 0.001). Exposure to 13 and 39 mg/kg diazinon were significantly different to 0 mg/kg (*P* < 0.001) and the dorsal raphe nucleus (DRN) was significantly different to all other brain regions (*P* < 0.001) (Bonferroni *post hoc* tests). p < 0.05 compared to all (#) other brain regions (one-way ANOVA (tissue as fixed factor) with Bonferonni *post hoc* tests for each dose) * p < 0.05 compared to 0 mg/kg (one-way ANOVA (dose as fixed factor) with Bonferonni *post hoc* tests for each region). Mean ± SEM. Cerebellum (CB), caudate putamen (CP), hippocampus (HP), prefrontal cortex (PFC).

|  | Time | | Time*Dose | | Dose | | Bonferonni comparisons  with 0 mg/kg, *P* | | |
| --- | --- | --- | --- | --- | --- | --- | --- | --- | --- |
|  | *F (df)* | *P* | *F (df)* | *P* | *F (df)* | *P* |  |  |  |
|  |  |  |  |  |  |  | 1 mg/kg | 13 mg/kg | 39 mg/kg |
| Blood | 17.5 (2,76) | **.000** | 2.5 (6,76) | **.030** | 2.0 (3,76) | .123 |  |  |  |
| Cerebellum | 2.9 (2,71) | .061 | 0.7 (6,71) | .650 | 0.9 (3,71) | .446 |  |  |  |
| Caudate Putamen | 0.6 (2,75) | .574 | 1.6 (6,75) | .150 | 7.6 (3,75) | **.000** | 1.000 | .288 | **.001** |
| Hippocampus | 8.5 (2,73) | **.000** | 1.7 (6,73) | .129 | 3.1 (3,73) | **.030** | 1.000 | **.025** | 1.000 |
| Prefrontal cortex | 2.9 (2,75) | .061 | 1.3 (6,75) | .274 | 4.2 (3,75) | **.008** | 1.000 | **.027** | **.039** |
| Dorsal raphe nucleus | 1.0 (2,70) | .360 | 0.8 (6,70) | .565 | 17.6 (3,70) | **.000** | .107 | **.000** | **.000** |

**Supplementary Table 1**  Univariate general linear model test results showing the effect of time and dose of acute diazinon exposure (i.p.) on adult rat butrylcholinesterase activity in different tissues. If there was an effect of dose Bonferonni *post hoc* comparisons were made. *P* < 0.05 in bold.

**Supplementary Figure 2.** Inhibitory response of rat dorsal raphe neurones in *in vitro* slices to 25 µM 5-HT, one day after acute *in vivo* exposure to vehicle or diazinon (39 mg/kg i.p.). The reduction in the inhibitory response to 2 minute application of 5-HT following diazinon exposure approached significance (vehicle *n* = 23, diazinon *n* = 20; U = 154, p = 0.06; quartiles ± min/max).
